# Brightening and Directionality Control of Dark Excitons through Quasi-Bound States in the Continuum

**DOI:** 10.3390/nano13233028

**Published:** 2023-11-27

**Authors:** Sebastian Klimmer, Giancarlo Soavi, Isabelle Staude, Ángela Barreda

**Affiliations:** 1 Institute of Solid State Physics, Friedrich Schiller University Jena, 07743 Jena, Germany; 2 ARC Centre of Excellence for Transformative Meta-Optical Systems, Department of Electronic Materials Engineering, Research School of Physics, The Australian National University, Canberra, ACT 2601, Australia; 3 Abbe Center of Photonics, Friedrich Schiller University Jena, 07745 Jena, Germany; 4 Institute of Applied Physics, Friedrich Schiller University Jena, 07745 Jena, Germany; 5 Group of Displays and Photonics Applications, Carlos III University of Madrid, Avda. de la Universidad, 30, Leganés, 28911 Madrid, Spain

**Keywords:** 2D materials, dark excitons, metasurfaces, Purcell factor, directionality

## Abstract

Thanks to their long lifetime, spin-forbidden dark excitons in transition metal dichalcogenides are promising candidates for storage applications in opto-electronics and valleytronics. To date, their study has been hindered by inefficient generation mechanisms and the necessity for elaborate detection schemes. In this work, we propose a new hybrid platform that simultaneously addresses both challenges. We study an all-dielectric metasurface with two symmetrically protected quasi-bound states in the continuum to enhance both the excitation and emission of dark excitons in a tungsten diselenide monolayer under normal light incidence. Our simulations show a giant photoluminescence signal enhancement (∼520) along with directional emission, thus offering distinct advantages for opto-electronic and valleytronic devices.

## 1. Introduction

The growing demand for digital and cloud services requires a paradigm shift in the way we process information. Optics and photonics offer energy-efficient approaches [1,2] to increase data transmission rates by orders of magnitude compared to electronics based on the recently proposed concepts and demonstrations of lightwave electronics [3,4,5] and ultrafast all-optical operations [6,7,8].

One particularly promising platform to perform all-optical operations is monolayer transition metal dichalcogenides (TMDs) [9]. Such materials are direct bandgap semiconductors [10] whose unique optical properties are largely determined by the extreme 2D spatial confinement and the resulting strong Coulomb interaction. In particular, light–matter interactions are dominated by excitons (strongly bound electron-hole pairs), which are stable at room temperature due to their high binding energy of several hundred meV [11,12,13]. Excitons can be further distinguished between optically allowed (“bright”) and optically forbidden (“dark”) [14]. To understand the existence of both species, a closer look at their band structure is necessary. The aforementioned direct band gap in TMD monolayers lies at the corners of the hexagonal Brillouin zone, the K and K’ points (or valleys), respectively, which are energetically degenerate but nonequivalent due to their different selection rules [15]. The strong spin–orbit coupling results in massive spin splitting in the valence band (on the order of several hundred meV) and much smaller splitting in the conduction band (a few tens of meV) [13]. To fulfill spin–momentum conservation, only transitions between bands with the same dominant spin direction are optically allowed (bright) in the electric dipole approximation and with emission in the out-of-plane direction. Such bright excitons possess short lifetimes (a few ps) and are therefore ideal for sensing applications or fast light-emitting diodes [16,17,18]. In contrast, spin-forbidden dark excitons have a much longer lifetime and coherence time [19], as radiative recombination can only occur through a spin-flip [20], leading to the possibility of manipulating the system’s valley degree of freedom numerous times. The control of this spin-valley degree of freedom is a prerequisite for all-optical valleytronic devices [21] and holds great promise for all-optical information processing. However, the generation and detection of spin-forbidden dark excitons is challenging due to their zero in-plane dipole moment [22,23]. In recent years, several endeavors have been undertaken to achieve the brightening of dark excitons. Conventional approaches involve the application of strong in-plane magnetic fields [24,25], out-of-plane polarized surface plasmon polaritons [26], or the injection of out-of-plane polarized light [27]. These methods primarily amplify the excitation of dark excitons, whereas their efficient detection remains a problem due to the out-of-plane dipole-like radiation pattern that they exhibit. Current research focuses on employing objectives with large numerical apertures (NA) to tackle this issue [27,28].

Alternatively, all-dielectric metasurfaces with high refractive indices have emerged as a promising platform to address both challenges simultaneously. Particularly, bound states in the continuum [29,30] (BICs), resonances with theoretically infinite quality factors (*Q*-factors), offer a straightforward way to tailor the light–matter interaction of various all-dielectric structures. By breaking the symmetry of the system, a radiative channel can be opened, which manifests itself as a leaky mode with a high but finite *Q*-factor, also called ‘quasi-BIC’. This can be realized by metasurfaces consisting of dielectric meta-atoms with broken in-plane inversion symmetry, where the *Q*-factor depends on the asymmetry of the unit cell. In addition, their successful combination with TMDs has also been shown recently, e.g., for second-harmonic generation enhancement [31,32].

In a previous work [28], the combination of a TMD with a suspended photonic crystal slab that supports BIC resonances was investigated, and brightening as well as the manipulation of the directionality of dark excitons was reported. However, the still-limited influence on the emission direction could not be solved satisfactorily by this approach, which still requires a large NA objective or a non-collinear detection scheme, thus preventing the use of conventional microscopy setups. In this work, we propose an all-dielectric metasurface where two symmetrically protected quasi-BIC modes are excited. These allow the simultaneous enhancement of excitation and emission of dark excitons in a tungsten diselenide (WSe_2_) monolayer while illuminating and collecting the emitted radiation perpendicular to the substrate, overcoming remaining technical problems in the study of dark excitons. We choose WSe_2_ based on the characteristic band ordering of tungsten-based TMDs, which is characterized by an energetically well-separated dark state associated with the spin-forbidden A exciton below the bright A exciton state [14,33]. Moreover, using WSe_2_ provides a direct comparison of the suggested dielectric metasurface with the results reported by Ma et al. [28]. Our results indicate a substantial increase in the photoluminescence signal (∼520), which is obtained as the product of the excitation and emission enhancement. We thus foresee a direct impact of our results on the experimental study of spin-forbidden dark excitons. The possibility of a massively simplified study of these quasiparticles could lead to an improved fundamental understanding of their properties and further pave the way for applications in all-optical information processing.

## 2. Materials and Methods

- Transmission spectra and near-field maps: The simulations were conducted by means of the finite-difference time-domain (FDTD) method, implemented in the software Ansys Lumerical 2023 [34]. For the transmission spectra, a unit cell of the metasurface was simulated and periodic boundary conditions were applied in the *x*- and *y*-axes. In the *z*-axis, perfectly matched layers (PML) were considered. The structure was illuminated by a plane wave source linearly polarized along the *x*- and *y*-axes and propagating at normal incidence from the top of the metasurface. The two nanobars conforming the unit cell rest on a semi-infinite silica substrate. A monitor in the silica substrate was used to obtain the transmission spectrum. The electric field intensity near-field maps were obtained through a frequency-domain field and power monitor in the ZX-plane at y=0. The electric field vectors were represented using Matlab. Auto non-uniform mesh (mesh accuracy 4) was selected. To ensure the convergence of the results, a finer mesh of 2 nm was chosen for the nanoresonators.
- Polarization vectors of the E-field. The polarization vectors of the E-field in the ZX-plane were obtained using the frequency-domain solver of the software CST Studio Suite 2019 [35]. Similarly to the calculations with Lumerical, a unit cell of the metasurface was simulated with periodic boundary conditions (unit cell boundaries). In the *z*-axis, open conditions were used. The number of Floquet modes selected for the Z${}_{\max}$ and Z${}_{\min}$ ports was 10. To visualize the polarization vectors of the E-field represented by arrows in the ZX-plane, field monitors were chosen at the resonance wavelength.
- Excitation enhancement: The excitation enhancement of the out-of-plane excitons was estimated through the near-field maps obtained with Ansys Lumerical [34] as described in the section Transmission spectra and near-field maps. We calculated the average intensity enhancement of the electric field on the XY-plane at a distance d=10 nm above the resonators. This distance was chosen to account for the hBN (hexagonal boron nitride) encapsulation of the 2D material seated on the top of the metasurface (XY-plane). The electric field intensity was obtained considering only the *z*-component of the electric field ($|E_{z}{|}^{2}$) due to the orientation of the excitons. For the normalization, $|E_{z}{|}^{2}$ was divided by the electric field intensity without the metasurface ($|E_{0}{|}^{2}|$), $<|E_{z}{|}^{2}/{\left|E_{0}\right|}^{2}>$.
- Emission enhancement: The emission enhancement was attained using the reciprocity principle following the method described in [36]. These simulations were conducted with the software COMSOL Multiphysics 6.0 [37], which is based on the finite element method. A unit cell of the metasurface was simulated, and periodic boundary conditions were used along the *x*- and *y*-axes. Perfectly matched layers were considered along the *z*-axis. The structure was illuminated with a linearly polarized plane wave by means of a port located in the air layer. The angles of incidence were swept from θ=0° to θ=24° (angles corresponding to a numerical aperture of NA ≈0.4) and from ϕ=0° to ϕ=180°. For each angle of incidence, the average of the electric field intensity, considering only the *z*-component of the electric field $E_{z}$, on a surface 10 nm above the top of the resonators was calculated. This surface represents the area where the out-of-plane dark excitons are randomly located. The sum of the average electric field intensity for each angle of incidence and for both polarizations of the incident radiation (TE and TM polarizations) was normalized to the emission enhancement for the case of a bare silica substrate $P_{0}$ (see Equation (1)).
  (1) $$G_{\mathrm{ED}}=\frac{1}{P_{0}}\sum_{\theta ,\phi ,\mathrm{TE},\mathrm{TM}}<|E_{z}{\left(r;\theta ,\phi \right)|}^{2}>$$
  Note that if the apodization factor is considered, Equation (1) must be multiplied by sin(θ).
- The back focal plane maps were simulated with the finite element method implemented in the software COMSOL Multiphysics 6.0 [37]. These calculations were conducted using the reciprocity principle as described in the previous paragraph.

## 3. Results and Discussion

### 3.1. Design of the Metasurface

**Figure 1:**
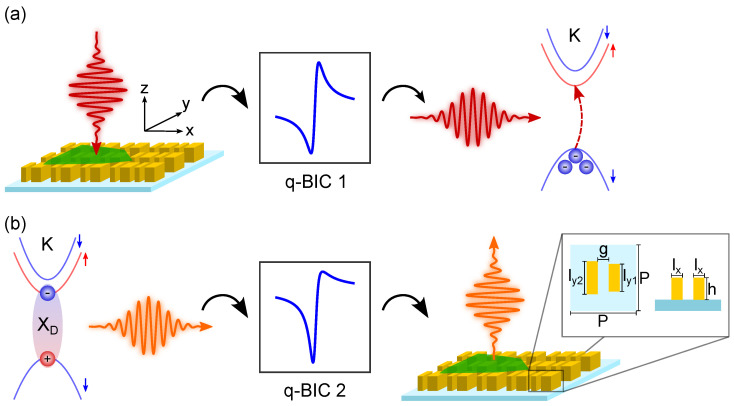
Schematic depiction (not to scale) of the excitation and emission of dark excitons in the proposed structure. (**a**) A normal incident light pulse (red) on the TMD/metasurface hybrid structure is converted to an out-of-plane polarized electric field by a quasi-BIC resonance (q-BIC 1). Thus, the light pulse is able to excite dark-excitons with electrons and holes located in bands with opposite spin at the K points of the Brillouin zone. Spin-up (-down) bands are marked with red (blue) arrows. (**b**) The spin-forbidden dark exciton (X${}_{D}$) recombines again radiatively through a spin-flip process. The emitted photoluminescence signal (orange) in the xy-plane is converted back to the z-direction by the second quasi-BIC resonance (q-BIC 2) of the metasurface. The inset shows a zoom in on the metasurface unit cell, where its geometric parameters are indicated.

As mentioned in the introduction, dark exciton emission involves spin-forbidden optical transitions with an out-of-plane dipole moment and are decoupled from radiative channels [22]. With the aim of enhancing the emission of the dark excitons and extracting the emitted radiation into the far-field, we design an all-dielectric silicon metasurface showing two resonances with out-of-plane electric field components at the excitation and emission wavelengths of the dark excitons: ($\lambda_{\mathrm{exc}}=733.3$ nm and $\lambda_{\mathrm{em}}=760.9$ nm, respectively). For instance, such out-of-plane quasi-BIC resonances have also been demonstrated on anisotropic plasmonic metasurfaces [38]. The working principle of the TMD/metasurface hybrid structure is schematically shown in Figure 1a,b.

**Figure 2:**
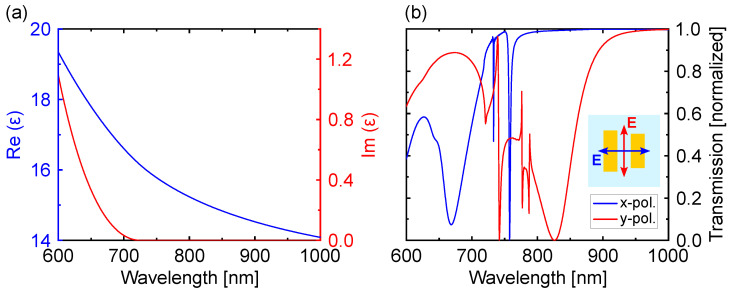
(**a**) Real (blue curve) and imaginary (red curve) parts of the dielectric permittivity ϵ of silicon obtained from ellipsometric measurements. (**b**) Transmission spectrum of the metasurface under illumination by a plane wave linearly polarized along the *x*-axis (blue curve) or *y*-axis (red curve) and propagating along the negative direction of the *z*-axis. The polarization direction with respect to the unit cell is shown in the inset.

The metasurface design is based on the concept of high-refractive-index (HRI) dielectric 2D lattices with asymmetric unit cells to excite quasi-BIC modes [32,39,40,41,42]. The metasurface unit cell consists of two amorphous silicon asymmetric nanobars of different widths along the *y*-axis, as shown in the inset of Figure 1b. The asymmetry parameter was chosen such that the *Q*-factors of the resonances and corresponding field enhancement values can be realistically targeted in experiments. In particular, the finite size of the sample, its roughness, and the small imperfections of the fabricated sample with respect to the numerical design decrease the *Q*-factor. The field size can be easily increased and is usually not the limiting factor. Instead, factors like roughness and small fabrication imperfections are much harder to control and determine the saturation value of *Q* in most experiments [43]. In previous works, by means of the design of metasurfaces composed of two asymmetric silicon nanobars, we were able to experimentally achieve *Q*-factors as large as 300 [44]. However, in theoretical works, it has been demonstrated that *Q*-factors larger than 3000 can be attained for quasi-BIC modes excited in all-dielectric metasurfaces [45]. The optical properties of silicon were obtained from ellipsometric measurements. In Figure 2a, the real and imaginary parts of the dielectric permittivity ϵ of silicon are depicted, whereas the imaginary part, responsible for the absorption, takes negligible values at the excitation and emission wavelengths of the dark excitons. The metasurface is located on a silica substrate, whose refractive index was fixed to $n_{s}=1.51$ in the analyzed spectral range. Due to the broken symmetry of the metasurface unit cell, it is possible to couple the dark out-of-plane polarized electric field with the field emitted by bright in-plane dipoles. This coupling produces a Fano-like behavior, allowing the excitation of the out-of-plane electric dipole moment of the metasurface with normal incidence light [46]. At the same time, by reciprocity, the out-of-plane electric dipole moment can emit radiation to the far-field in the zero-order transmission. By coupling the out-of-plane electric dipole moment of the metasurface with the excitons, it becomes possible to brighten the excitons and enhance their directional emission.

The dimensions of our design are as follows: silicon bar lengths $l_{y1}=230\;nm$ and $l_{y2}=217\;nm$, width $l_{x}=109\;nm$, height h=172 nm, and gap between the two bars g=98 nm. The period of the metasurface corresponds to P=476 nm (see inset of Figure 1b).

### 3.2. Transmission Spectra of the Metasurface

**Figure 3:**
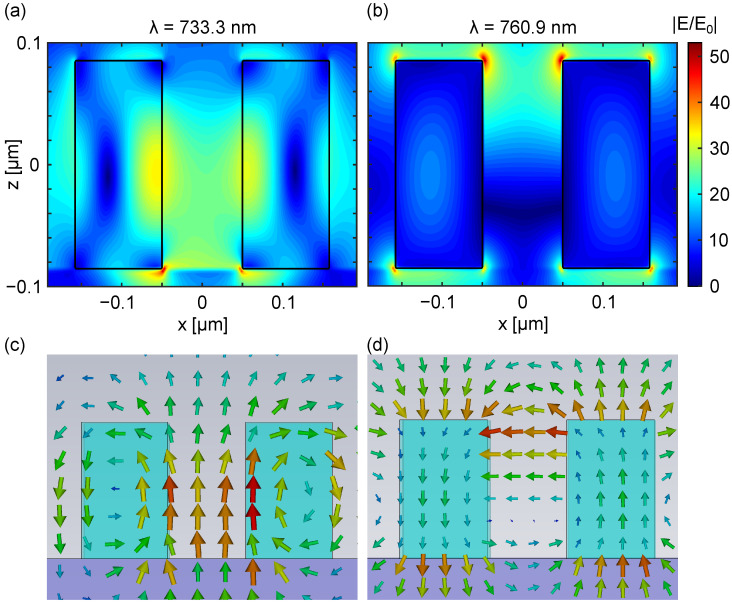
(**a**,**b**) Near-field electric field maps in one unit cell of the metasurface at λ=733.3 nm and λ=760.9 nm, respectively. (**c**,**d**) Polarization vectors of the electric field at λ=733.3 nm and λ=760.9 nm, respectively. The metasurface was illuminated with a plane wave linearly polarized along the *x*-axis and propagating along the negative direction of the *z*-axis. In all panels, the ZX-plane is shown intersecting the bars at their center, the edges of which are indicated by the black contours.

We have obtained the transmission spectra for the designed metasurface when it is illuminated with a plane wave linearly polarized along the *x*-axis (axis that joins both nanobars in the unit cell) and *y*-axis (axis along the asymmetry of the unit cell) under normal incidence (negative direction of the *z*-axis). In the transmission spectrum corresponding the the *x*-axis polarization, represented in Figure 2b in blue, we observe two narrow resonances at λ=733.3 nm and λ=760.9 nm (excitation and emission wavelengths of the dark excitons), showing a *Q*-factor of 4313 and 380, respectively. It is worth remarking that higher *Q*-factors could be obtained by decreasing the asymmetry between the two nanobars. However, we have kept this design to consider feasible dimensions from a fabrication point of view. For the polarization along the *y*-axis, no resonances can be observed at the excitation and emission wavelengths of the dark excitons (see red curve in the transmission spectrum in Figure 2b). For that reason, we focus on the *x*-axis polarization in the manuscript. By means of an analysis of the near-field profile of the modes excited for the *x*-axis polarization (Figure 3), we can identify both resonances in Figure 2b. At the excitation wavelength (λ=733 nm), the norm of the electric field enhancement shows an in-plane antiferromagnetic (AFM) order with an out-of-plane electric dipole (ED) field induced between the two nanobars. This antiferromagnetic mode refers to the characteristics of the magnetic near-fields excited in each one of the nanobars composing the unit cell of the metasurface, which are oriented in opposite directions [47]. At the emission wavelength (λ=760.9 nm), the norm of the electric field shows an out-of-phase coupled out-of-plane ED pair, which induces a magnetic dipole (MD) field between the two bars [47]. For the shorter wavelength resonance, the highest electric field is attained between the nanobars, whereas for the larger wavelength resonance, the highest electric field occurs at the top of the resonators, where the 2D material would be located. The first resonance is responsible for excitation rate enhancement, and the second one contributes to enhance dark exciton emission and its collection in the upward direction.

### 3.3. Photoluminescence Signal Enhancement

The photoluminescence (PL) signal enhancement of a single emitter located at the position $r_{\mathrm{em}}$ due to the presence of a nanostructure is proportional to the gains in the excitation rate ($\Gamma_{\mathrm{exc}}\left(r_{\mathrm{em}},\lambda_{\mathrm{exc}}\right)$), the quantum yield ($\mathrm{QY}(r_{\mathrm{em}},\lambda_{\mathrm{em}})$), and the collection efficiency ($D(r_{\mathrm{em}},\lambda_{\mathrm{em}})$) through the following equation [48,49]:

(2) $$\mathrm{EF}\propto \frac{\Gamma_{\mathrm{exc}}\left(r_{\mathrm{em}},\lambda_{\mathrm{exc}}\right)}{\Gamma_{\mathrm{exc}}^{0}}\;\cdot \;\frac{\mathrm{QY}(r_{\mathrm{em}},\lambda_{\mathrm{em}})}{{\mathrm{QY}}^{0}}\;\cdot \;\frac{D(r_{\mathrm{em}},\lambda_{\mathrm{em}})}{D^{0}}$$

where $\lambda_{\mathrm{exc}}$ and $\lambda_{\mathrm{em}}$ refer to the excitation and emission wavelengths, respectively. $\Gamma^{0}$, ${\mathrm{QY}}^{0}$, and $D^{0}$ are the excitation rate, quantum yield, and collection efficiency in the absence of the nanostructure, respectively. EF stands for fluorescence enhancement.

The excitation rate is proportional to the local enhancement of the incident intensity at the position where the emitter is located [50]:

(3) $$\frac{\Gamma_{\mathrm{exc}}\left(r_{\mathrm{em}},\lambda_{\mathrm{exc}}\right)}{\Gamma_{\mathrm{exc}}^{0}}=\frac{|E\left(r_{\mathrm{em}},\lambda_{\mathrm{exc}}\right){|}^{2}}{|E_{0}{|}^{2}}$$

where $|E_{0}|$ is the amplitude of the incident electric field, and $\left|E(r_{\mathrm{em}},\lambda_{\mathrm{exc}})\right|$ is the local electric field strength at the position of the emitter $r_{\mathrm{em}}$.

For low quantum yield emitters, the gain in the quantum yield $\mathrm{QY}\left(r_{\mathrm{em}},\lambda_{\mathrm{em}}\right)/{\mathrm{QY}}^{0}$ can be expressed as:

(4) $$\frac{\mathrm{QY}(r_{\mathrm{em}},\lambda_{\mathrm{em}})}{{\mathrm{QY}}^{0}}\approx \frac{\Gamma_{r}\left(r_{\mathrm{em}},\lambda_{\mathrm{em}}\right)}{\Gamma_{r}^{0}}$$

where $\Gamma_{r}\left(r_{\mathrm{em}},\lambda_{\mathrm{em}}\right)$ and $\Gamma_{r}^{0}$ are the radiative decay rate in the presence or absence of the nanostructure, respectively [49].

The collection efficiency or directionality is the ratio of the collected signal into a given numerical aperture (NA) and the total radiated power in free space $P_{\mathrm{rad}}^{\mathrm{out}}$ [51]

(5) $$D\left(r_{\mathrm{em}},\lambda_{\mathrm{em}}\right)=\frac{1}{P_{\mathrm{rad}}^{\mathrm{out}}}\underset{\mathrm{NA}}{\;\int \int \;}P\left(\theta ,\phi \right)\sin\left(\theta \right)d\theta d\phi$$

P(θ,ϕ) is the angular power density radiated in free space along the direction defined by the polar and azimuthal angles θ and ϕ.

The gain in the directivity can be expressed as $D\left(r_{\mathrm{em}},\lambda_{\mathrm{em}}\right)/D^{0}$, where $D(r_{\mathrm{em}},\lambda_{\mathrm{em}})$ and $D^{0}$ are the directivity in the presence or absence of the nanostructure, respectively.

From this description, we can define the PL signal enhancement as the product of the excitation and emission enhancement [52]:

(6) $$\mathrm{EF}\approx \eta_{\mathrm{exc}}\left(r_{\mathrm{em}},\lambda_{\mathrm{exc}}\right)\;\cdot \;\eta_{\mathrm{em}}\left(r_{\mathrm{em}},\lambda_{\mathrm{em}},\mathrm{NA}\right)$$

where $\eta_{\mathrm{exc}}\left(r_{\mathrm{em}},\lambda_{\mathrm{exc}}\right)=\frac{\Gamma_{\mathrm{exc}}\left(r_{\mathrm{em}},\lambda_{\mathrm{exc}}\right)}{\Gamma_{\mathrm{exc}}^{0}}$ and $\eta_{\mathrm{em}}=\frac{\Gamma_{r}\left(r_{\mathrm{em}},\lambda_{\mathrm{em}}\right)}{\Gamma_{r}^{0}}\;\cdot \;\frac{D(r_{\mathrm{em}},\lambda_{\mathrm{em}})}{D^{0}}$

In order to attain the PL signal enhancement, in the following sections we analyze the excitation and emission enhancement.

### 3.4. Excitation Enhancement

**Figure 4:**
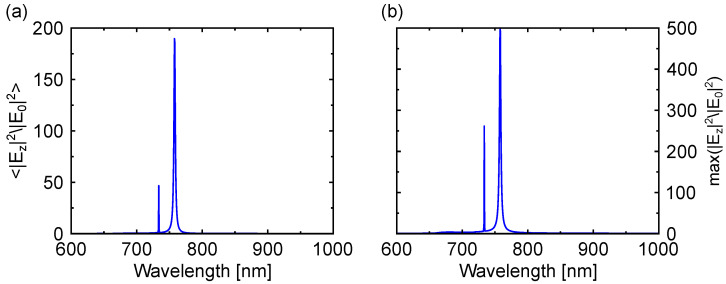
(**a**) Average intensity enhancement of the *z*-component of the electric field ($<|E_{z}{|}^{2}/{\left|E_{0}\right|}^{2}>$) at the XY-plane at a distance from the top of the metasurface of d=10 nm. (**b**) Maximum intensity enhancement of the *z*-component of the electric field ($\mathrm{Max}(|E_{z}{|}^{2}/|E_{0}{|}^{2})$) at the XY-plane at a distance from the top of the metasurface of d=10 nm.

The excitation rate, which can be defined as the rate transition between different energy levels induced by the excitation of incident radiation at a certain wavelength $\lambda_{\mathrm{exc}}$, is proportional to the local intensity enhancement of the incident radiation at the position of the emitter [53]. Taking into account that the 2D material will be seated at the top surface of the nanobars composing the metasurface, we have calculated the electric field intensity enhancement on a surface at d=10 nm from the top of the nanobars. The reason for having the distance between the top surface of the metasurface and the surface plane where the simulations are performed is to consider the fact that the 2D material will be bottom-encapsulated with hBN to avoid possible charge transfer from the silicon metasurface to the TMD, which would result in a spectral shift of the PL emission [54,55]. At that surface, we have obtained the average (Figure 4a) and maximum (Figure 4b) intensity enhancement of the *z*-component of the electric field. The average has been used with the aim to take into account the different positions of the dipoles in the metasurface. As our objective is to enhance the excitation of out-of-plane dipole moments, we just consider the *z*-component of the electric field in the calculations. However, it should be noted that for the case of randomly oriented dipoles, the average intensity enhancement must include all the components of the electric field to account for all the possible dipole orientations. From Figure 4, we observe that the intensity enhancement of the *z*-component of the electric field is the largest at the quasi-BIC resonances, which correspond to the excitation and emission wavelengths of the out-of-plane excitons. According to the description in the PL signal enhancement section, we are interested in the average intensity enhancement at the excitation wavelength (Figure 4a), which corresponds to $<|E_{z}{|}^{2}/{\left|E_{0}\right|}^{2}>=46.63$.

### 3.5. Emission Enhancement

The emission of quantum emitters can be enhanced by modifying the electromagnetic environment where the emitters are located via the local density of optical states (LDOS) [56]. The LDOS quantifies the available amount of electromagnetic states that can be occupied by a photon in a certain position of a system, and at resonance, it gives the Purcell factor. The Purcell factor accounts for the enhancement of spontaneous emission of an emitter due to the optical states compared to a homogeneous background [57,58,59,60,61]. The near-field intensity distribution in the surroundings of the nanoparticles determines the LDOS [62,63]. Specifically, strong electromagnetic energy concentrations in small volumes lead to high Purcell factors [64,65]. In the Purcell factor calculations, a dipole is considered as the illumination source. In the case of working with periodic structures like metasurfaces, the dipolar source combined with periodic boundary conditions suggests infinite dipolar sources, which is not consistent with the incoherent emission of randomly distributed quantum emitters.

As a solution, in some previous works, the emission enhancement of randomly oriented and homogeneously distributed emitters on a metasurface was attained by means of the reciprocity principle [36]. In this case, a unit cell of the metasurface is simulated, and periodic boundary conditions are considered. The emission enhancement can be achieved by means of the spatial average of the local electric field intensity excited by a plane wave that incidences at a certain polar and azimuthal angle over the active part where the emitters are located. The average electric field intensity enhancement is summed for all the possible polar and azimuthal angles and both polarizations of the incident radiation (TE and TM). The result is normalized to the emission without considering the nanoantennas conforming the metasurface. In our work, the emitters are not randomly oriented, as we are interested in the out-of-plane excitons (dipoles oriented along the *z*-axis). However, we can still use the reciprocity principle considering only the *z*-component of the local electric field to calculate the emission enhancement (see ‘Methods’ for the simulation details).

Due to the thickness of the 2D material (<1 nm), the active part is a surface the size of the unit cell and is located 10 nm above the resonators, which corresponds to the emitters’ position. In order to take into account the experimental conditions, in the summation of Equation (1), only the emission within the solid angles that correspond to a NA = 0.4 objective are considered. Following the described procedure, we have obtained an average emission enhancement of approximately 11 over a 0.4 NA. This value is almost equal to that reported in [28] corresponding to 12. However, in that work, the emission enhancement is obtained at non-normal incidence, increasing the complexity of the detection experimental setup. From the gains in the excitation and emission enhancement, we estimate an average PL signal enhancement of 522. In [28] an even larger PL signal enhancement (by a factor of 1400) was observed recently. Nevertheless, the reported emission angle of the dark exciton is about 48° with respect to the surface normal, which requires a large NA (≥0.75) objective or a non-collinear detection scheme.

### 3.6. Back Focal Plane Images

One of the factors contributing to the PL signal enhancement is the collection efficiency. With the aim to achieve superior collection efficiency, it is essential to design a metasurface that enables emission into a very low numerical aperture (NA), which is critical for many different applications such as single-photon sources. For that reason, we have considered in our design the excitation of a quasi-BIC resonance at the exciton emission wavelength. As mentioned, the dark excitons cannot be excited by a plane wave propagating under normal incidence. According to the reciprocity principle, this means that dark excitons cannot emit in the normal direction to the substrate plane. However, due to the coupling of the dark excitons to the out-of-plane electric dipole moments of the metasurface, the dark excitons are able to emit into the far field, with their emission mostly normal out of the sample plane. Specifically, the dark exciton radiation is coupled to the out-of-plane electric dipole moments, which are dark modes (BIC modes) for a symmetric metasurface. Due to the broken symmetry of the metasurface unit cell, the BIC modes are transformed into quasi-BIC modes, which couple the radiation of the out-of-plane electric dipole moments with the bright in-plane dipoles. The emission of the in-plane dipoles is mostly out-of-plane. In particular, the quasi-BIC resonance is characterized by a high *Q*-factor and a narrow emission pattern close to the direction perpendicular to the substrate, allowing the enhancement and directional emission of the dark excitons. However, in momentum space, most modes have a parabola-shaped dispersion. Hence, in order to obtain the out-of plane emission, the surface lattice resonance condition (SLR, around the Wood anomaly) can be used, with the wavelength in the substrate material approximately matching the lattice constant [66,67]. In our work, we are close to that condition, which should be attained at λ=719 nm, taking into account that P=476 nm and the substrate refractive index is n=1.51.

**Figure 5:**
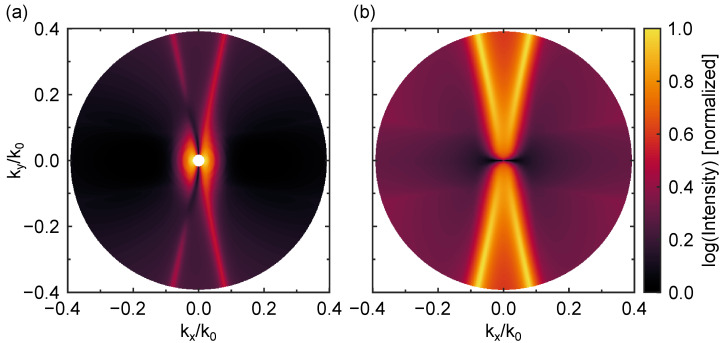
Back focal plane images at the dark exciton emission wavelength (λ=760.9 nm) corresponding to the emission of out-of-plane emitters (oriented along the *z*-axis) and randomly located at different positions of the XY-plane at a distance of d=10 nm above the top of the metasurface. (**a**,**b**) Depictions of the cases of polarization of the incident radiation TM and TE (i.e., for θ=0° and ϕ=0°; the polarization is along the axis that joins both nanoparticles of the unit cell or is perpendicular to it, respectively). The angular range corresponds to a numerical aperture of NA = 0.4. The colorbar shows the normalized logarithmic intensity, calculated from the *z*-component of the electric field.

In Figure 5, we show the back focal plane images at the exciton emission wavelength (λ=760.9 nm) corresponding to the emission of randomly out-of-plane dipoles (oriented along the *z*-axis) and located at different positions of the XY-plane at a distance of d=10 nm above the top of the metasurface. The back focal plane image is represented for both polarizations of the incident radiation: TM and TE (i.e., for θ=0° and ϕ=0°; the polarization is along the axis that joins both nanoparticles of the unit cell or is perpendicular to it, respectively). We map in the back focal plane image an angular range corresponding to the entire numerical aperture of a possible collection objective (NA = 0.4). As can be observed, most of the radiation is emitted within a narrow cone around the substrate normal.

**Figure 6:**
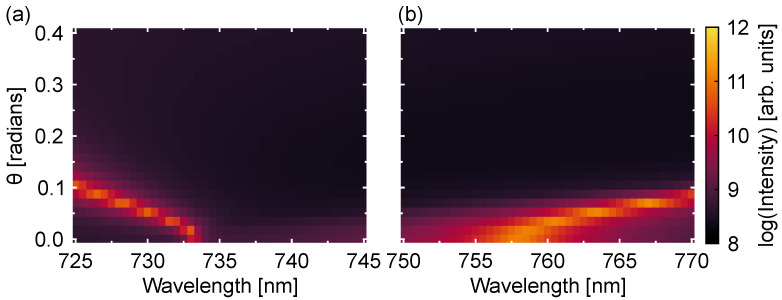
Angle-dependent intensity spectra for *x*-axis polarized light at the spectral regions corresponding to the (**a**) excitation and (**b**) emission wavelengths of the dark excitons. The intensity is calculated from the *z*-component of the electric field at a plane 10 nm above the metasurface and is represented in logarithmic scale. The angle ϕ is fixed to ϕ=0°.

To gain further insight by means of the momentum-resolved emission at the spectral ranges corresponding to the excitation and emission wavelengths of the dark excitons (Figure 6a,b, respectively), we have analyzed the emission patterns at different wavelengths. Specifically, it is possible to notice sharp dispersive resonances around λ=733 nm (see Figure 6a) and λ=761 nm (see Figure 6b), which are due to the excitation of out-of-plane quasi-BIC modes. The excitation of these resonances at normal incidence (TM polarization) puts into evidence the importance of choosing an appropriate lattice constant for the metasurface in order to improve the excitation and emission efficiency of the dark excitons in the 2D material.

## 4. Conclusions

In this work, we have investigated the PL signal enhancement of dark excitons of WSe_2_. As dark exciton emission involves spin-forbidden optical transitions with an out-of-plane dipole moment, it does not emit radiation to the far-field, and by the reciprocity principle, it cannot be excited by far-field illumination. With the aim of enhancing the emission of dark excitons and extracting the emitted radiation into the far-field, we have designed a symmetry-broken all-dielectric silicon metasurface showing two symmetry-protected quasi-BIC resonances at the excitation (λ=733 nm) and emission (λ=760.9 nm) wavelengths of the dark excitons. Both quasi-BIC modes correspond to out-of-plane electric dipole moments, and they can be excited at normal incidence. By means of the resonance at the excitation wavelength, it is possible to enhance the excitation of the dark excitons, obtaining an average excitation enhancement of 47. From the quasi-BIC mode at the emission wavelength of the dark excitons, we demonstrate an average emission enhancement of more than one order of magnitude, considering a numerical aperture of NA = 0.4. With this, we estimate an average PL signal enhancement due to the metasurface of 522. In terms of fabrication, the design of the proposed metasurface can be realized experimentally with a sufficiently high *Q*-factor, as has already been shown with similar geometries [44]. The subsequent transfer of a TMD monolayer can also be achieved analogously to previous works on comparable hybrid structures [31,32]. Thus, we anticipate that the experimental functionality of the suggested device will be consistent with our simulations. Therefore, our results provide a realistic guide for the design of more efficient devices based on dark excitons in atomically thin materials with possible applications in valleytronics and opto-electronics.

## Data Availability

Data underlying the results presented in this paper are not publicly available at this time but may be obtained from the authors upon reasonable request.
